# Epidemiology of Tennis-Related Injuries Among Competitive Youth Players in Tunisia: Frequency, Characteristics, and Management Patterns

**DOI:** 10.3390/medicina61081478

**Published:** 2025-08-18

**Authors:** Saoussen Layouni, Ismail Dergaa, Hela Ghali, Halil İbrahim Ceylan, Valentina Stefanica, Marwa Neguez, Ines Loubiri, Wissem Dhahbi, Chaima Rjiba, Sarra Ksibi, Sahbi Elmtaoua, Sonia Jemni, Helmi Ben Saad

**Affiliations:** 1 Faculty of Medicine of Sousse, University of Sousse, Sousse 4000, Tunisia; layouni.saoussen@famso.u-sousse.tn (S.L.); hela.ghali@famso.u-sousse.tn (H.G.); loubiri.iness@gmail.com (I.L.); sahbi.elmtaoua@famso.u-sousse.tn (S.E.); sonia.jemni@famso.u-sousse.tn (S.J.); 2 Physical Medicine and Rehabilitation Department, University Hospital Sahloul, Sousse 4054, Tunisia; 3 High Institute of Sport and Physical Education of Ksar Said, University of Manouba, Manouba 2010, Tunisia; phd.dergaa@gmail.com; 4 Department of Preventive and Community Medicine, University Hospital Sahloul, Sousse 4054, Tunisia; 5 Physical Education of Sports Teaching Department, Faculty of Kazim Karabekir Education, Ataturk University, Erzurum 25030, Türkiye; 6 Department of Physical Education and Sport, Faculty of Sciences, Physical Education and Informatics, National University of Science and Technology Politehnica Bucharest, Pitesti University Center, 060042 Pitesti, Romania; 7 Department of Family Medicine, Farhat HACHED Hospital, University of Sousse, Sousse 4031, Tunisia; neguezmarwa21@gmail.com (M.N.); chaimarjiba@gmail.com (C.R.); ksibisarra@yahoo.fr (S.K.); 8 Research Unit “Sport Sciences, Health and Movement”, Higher Institute of Sports and Physical Education of Kef, University of Jendouba, El Kef 8100, Tunisia; wissem.dhahbi@gmail.com; 9 Training Department, Police College, Qatar Police Academy, Doha 7157, Qatar; 10 Physical Medicine and Rehabilitation Department, University Hospital Ibn Jazzar, Kairouan 3100, Tunisia; 11 Heart Failure Research Laboratory (LR12SP09), Farhat HACHED Hospital, University of Sousse, Sousse 4000, Tunisia; helmi.bensaad@rns.tn

**Keywords:** adolescent athletes, tennis player, biomechanical stress, injury prevention, musculoskeletal trauma, sports medicine, subsequent injuries, youth sport specialization

## Abstract

*Background and Objectives*: Tennis involves repetitive high-velocity movements, rapid directional changes, and challenging environmental conditions, exposing players to injury risk. However, injury surveillance data for North African youth players are lacking. This study aimed to determine the frequency, characteristics, and management of tennis-related injuries among competitive Tunisian youth players. *Materials and Methods:* A cross-sectional study was conducted among players aged 5–18 years from tennis clubs (October 2023–November 2024). Data were collected using researcher-administered questionnaires, incorporating the Oslo Sports Trauma Research Center Overuse Injury Questionnaire and a sport-specialization assessment, following International Olympic Committee guidelines. *Results:* Among 256 players, 53.5% (*n* = 137) reported 366 injuries. Lower limbs were most affected (58.5%), followed by upper limbs (32.8%); knees (23.2%), ankles (17.5%), and wrists (10.1%). Muscle/tendon (36.9%), superficial tissue (28.1%), and ligament/joint capsule injuries (27.6%) predominated. Most injuries occurred during practice (74.9%) and hot weather (93.4%). Severe injuries represented 24%, while 29.5% were minor without time loss. Subsequent injuries occurred in 54.6% of injured players, with significantly higher rates in those with incomplete rehabilitation (*p* < 0.001). *Conclusions*: The high frequency of recurrent injuries and limited rehabilitation highlight critical gaps in injury management, emphasizing the need for targeted neuromuscular training, accessible rehabilitation, and standardized return-to-play protocols.

## 1. Introduction

Tennis is one of the most popular individual sports worldwide, with over 87 million players, including around 33 million under 18 years of age [1,2]. In Tunisia, the number of registered players increased to 18,500 in 2021 (0.16% of the population), a fourfold rise since 2018 [1]. This growth has been fueled by the international success of players such as Ons Jabeur, the first Arab and African woman to reach consecutive Wimbledon finals (2022–2023) [3,4]. The increasing visibility of Tunisian tennis has encouraged more children and adolescents to engage in both recreational and competitive play.

Tennis presents unique physical demands on the developing musculoskeletal system of youth players [3]. Unlike many other sports, tennis matches have no predetermined time limits and can last up to five hours, requiring high levels of both aerobic endurance (70–80% of energy contribution) and anaerobic power (10–20% of energy contribution) for explosive movements [4,5]. The sport involves repetitive high-velocity arm movements (reaching speeds of 1700°/s during serves) combined with frequent high-intensity movements, including 300–500 explosive bursts during a typical match and directional changes every 4–10 s [6,7]. These movements generate forces reaching 5–10 times body weight throughout the entire kinetic chain, exposing youth players with immature musculoskeletal systems to both acute traumatic injuries and chronic overuse conditions [3,8]. The repetitive nature of tennis strokes, combined with the high forces generated during serves and groundstrokes, places particular stress on the upper extremities. In contrast, the lower extremities are subjected to considerable loading during rapid directional changes [9]. Youth tennis players are particularly vulnerable during periods of growth and maturation, when skeletal growth outpaces muscular development, creating tendinous tightness and potential biomechanical imbalances [10].

Despite the growing popularity of tennis in Tunisia and the recognized injury risks associated with the sport, significant research gaps exist in understanding the epidemiology of tennis injuries among youth players. While several studies have investigated tennis injuries in adult players [11] and elite junior players in Western countries [2,12], a notable paucity of research remains regarding tennis injuries among youth players in North Africa. To the authors’ knowledge, no thorough epidemiological studies have been conducted in this region to assess the prevalence, types, and management of these injuries, despite the sport’s growing popularity and distinct playing conditions. The specific characteristics of tennis injuries in this population, including frequency, anatomical distribution, severity, and recovery patterns, remain largely unexplored. Furthermore, the unique environmental conditions in Tunisia, including ambient temperatures frequently exceeding 35 °C during the summer months and predominantly hard-court surfaces (accounting for over 85% of playing facilities), likely influence injury patterns in ways that differ significantly from those observed in temperate regions [13,14]. Additionally, differences in access to specialized sports medicine resources, coaching expertise, and structured injury prevention programs may further distinguish injury profiles in this population [12].

The anthropometric profiles for elite athletes from various sports during pre-season training will be a valuable resource for sports professionals when monitoring and interpreting body composition data. Considerable variation exists in anthropometric profiles among different athletes and sports, highlighting the need for sport-specific normative ranges to enable optimal monitoring of individual athletes, particularly those varying across sports, age, training status, and position [15]. Identifying region-specific injury patterns is essential for developing targeted prevention strategies that address the unique needs of Tunisian youth tennis players, ultimately supporting their long-term health and athletic development. Thus, this study aimed to determine the frequency and characteristics of tennis injuries among youth Tunisian players, including injury types, anatomical distribution, severity, circumstances, and management approaches.

## 2. Materials and Methods

### 2.1. Study Design

This study received approval from the Ethics Committee of the Faculty of Medicine in Sousse, Tunisia (approval reference number: CEFMS 203/2023). All participating players and their legal representatives provided oral informed consent. Confidentiality and anonymity were maintained throughout the study, with data stored securely according to institutional protocols. It also complied with the ethical and procedural requirements for conducting sports medicine and exercise science research.

### 2.2. Sample Size Calculation

The minimum sample size was calculated to estimate a 12.1% frequency (12) in a population of 2388 with 5% precision at 95% confidence. Assuming a design effect of 1, the required sample size was 154 participants, adjusted for finite population size. After accounting for a 20% anticipated loss to follow-up, the final sample size was 185. The calculation was performed using EPI Info Version 6.04 software.

### 2.3. Participants and Setting

This cross-sectional study followed the STROBE (Strengthening the Reporting of Observational Studies in Epidemiology) guidelines for cross-sectional studies [16]. It targeted all competitive youth tennis players aged 18 years and below enrolled in tennis clubs in the cities of Sousse, Monastir, and Mahdia in Tunisia. These three coastal cities represent major tennis centers in Tunisia, collectively accounting for approximately 35% of the country’s registered competitive junior players. The region features predominantly hard courts (92%) with limited clay court facilities (8%), reflecting the typical playing environment in the country.

**Figure 1:**
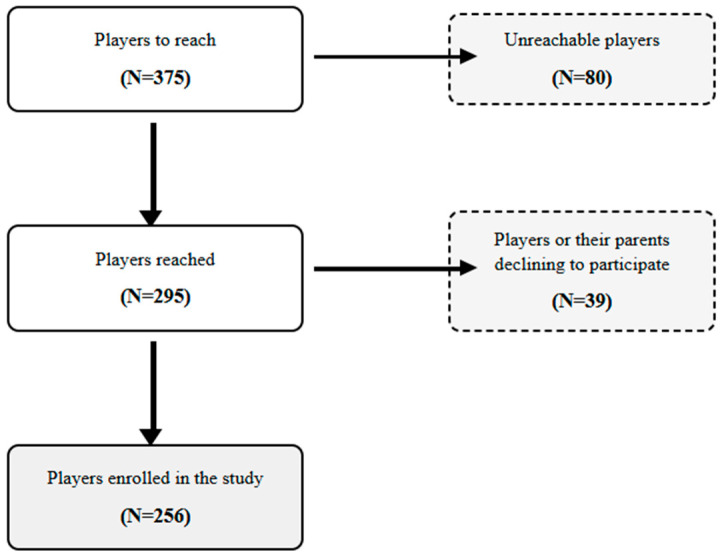
Flow chart of young tennis players’ recruitment between October 2023 and August 2024. A total of 375 players were initially contacted. Of these, 80 were unreachable, and 39 declined to participate, resulting in 256 enrolled players.

A total of 375 youth tennis players were initially contacted through club registries in Sousse, Monastir, and Mahdia. Of these, 80 players were unreachable due to outdated contact information or a lack of response. Among the 295 reachable players, 39 declined to participate (either the player or their legal guardian), resulting in a final sample of 256 participants enrolled in the study (Figure 1).

Inclusion criteria were: (i) age under 18 years; (ii) involvement in competitive tennis; (iii) regular participation in organized training (minimum 4 h/week) or competitions (minimum 10 matches annually); and (iv) consent from both the player and their legal representative. The inclusion criteria included the following: recreational players, regular participants, and those with proper consent. Players with a documented history of musculoskeletal conditions unrelated to tennis were excluded, as were those with incomplete questionnaires (less than 70% of questions answered).

### 2.4. Study Protocol and Data Collection Procedures

The study was conducted between October 2023 and November 2024, encompassing both competitive and non-competitive seasons to capture year-round injury patterns. The recruitment process is illustrated in Figure 1. After receiving approval from the administrative committees of the respective clubs, we implemented a systematic recruitment strategy. Eligible players and their parents were contacted through club registries to secure written informed consent for participation. Appointments were scheduled at the clubs during regular training sessions.

To standardize data collection, a pilot study was conducted with 15 players (not included in the final analysis) to refine the questionnaire, assess comprehension, and estimate completion time (approximately 25 min). All data were collected by a single trained sports medicine physician (MN) to ensure consistency. Questionnaires were administered via structured interviews with both the player and at least one legal representative present to minimize recall bias, particularly for younger participants and historical injury data.

Age was noted, and participants were distributed across six age categories as follows: Lutin (<8 years), Poussin (8–10 years), Benjamin (10–12 years), Minim (12–14 years), Cadet (14–16 years), and Junior (16–18 years). Anthropometric measurements were conducted using standardized procedures. Height was measured using a portable stadiometer (Seca 213, Hamburg, Germany) with a precision of 0.1 cm, and weight was measured using a calibrated digital scale (Tanita BC-545N, Tokyo, Japan) with a precision of 0.1 kg, with participants wearing light clothing and no shoes [15]. Body mass index (BMI) was calculated (kg/m^2^). The corpulence status was identified according to age and sex-specific WHO (World Health Organization) growth charts [17] as follows: underweight (BMI: <5th percentile), normal (BMI: 5th to 85th percentiles), overweight (BMI: 85th to 95th percentile), obese (BMI: >95th percentile). Pubertal status was determined through a direct question addressed to the athlete and/or their parent, asking whether the player had entered puberty (yes/no).

### 2.5. Assessment Instruments

#### 2.5.1. Data Collection Form

A comprehensive data collection instrument was developed based on a thorough literature review and expert consultation with sports medicine physicians, tennis coaches, and epidemiologists. The final questionnaire consisted of seven sections covering: (i) sociodemographic and medical data; (ii) sporting characteristics; (iii) sport specialization status; (iv) game environment; (v) injury details; (vi) The Oslo Sports Trauma Research Center Overuse Injury Questionnaire (OSTRC-02); and (vii) injury prevention practices. The form collected exposure data, including training hours per week, match play frequency, and playing surfaces.

For injury classification, we followed the International Olympic Committee consensus statement on methods for recording and reporting epidemiological data on injury and illness in sport [18] and its extension specific to tennis [19]. Athletes were asked to document all injuries sustained in their sporting history. Injuries were categorized by anatomical location, tissue type, mode of onset, mechanism, severity, and circumstances. The following injury severities were determined: minor with no time loss, mild (1–7 days’ time loss), moderate (8–28 days’ time loss), and severe injuries (>28 days’ time loss) [8,18].

#### 2.5.2. The OSTRC-O2 Injury Questionnaire

The validated OSTRC-O2 overuse injury questionnaire [20] was used to capture overuse injuries that might not be reported through traditional time-loss definitions. This instrument has demonstrated high internal consistency (Cronbach’s alpha = 0.91) and test-retest reliability (ICC = 0.88) in adolescent athletic populations [21]. This validated tool assesses the impact of overuse injuries on participation, training volume, performance, and pain. The severity score ranges from 0 (full participation without health issues) to 100 (no involvement due to health issues). To enhance injury recall accuracy, a visual body chart was used to identify anatomical locations, and reference to calendar events (such as tournaments and school holidays) was employed to establish the timing of injuries [22]. When available, medical records and imaging reports were reviewed to verify diagnoses. The OSTRC-O2 questionnaire identified overuse injuries in 22% of the study population. The problems were classified as mild (severity score: 1–30 points), moderate (severity score: 31–60 points), and severe (severity score: 61–100 points) [20].

#### 2.5.3. Sport Specialization Scale

The validated 3-point specialization scale developed by Jayanthi et al. [8] was used to categorize athletes based on their degree of specialization. This instrument evaluates the following three criteria: (i) year-round training (>8 months), (ii) exclusive participation in tennis, and (iii) quitting other sports to focus on tennis. Players with a score of three were classified as highly specialized, those with a score of two as moderately specialized, and those with scores of zero or one as lowly specialized.

### 2.6. Statistical Analysis

Statistical analysis was performed using SPSS software version 25.0 (IBM Corp., Armonk, NY, USA). For quantitative data, the normality of variables was verified using the Kolmogorov-Smirnov test. Normally distributed variables were presented as mean ± standard deviation, while non-normally distributed variables were represented by median and interquartile range (IQR). Categorical data were presented in terms of frequency and percentage. Descriptive statistics were used to characterize the study population and analyze injury patterns. The frequency of injuries was calculated as the percentage of players who reported at least one injury. We used Pearson’s chi-square test or Fisher’s exact test for percentage comparisons, including analyses of injury prevalence across age categories, associations between injury severity and medical care utilization, differences in muscle–tendon injury prevalence between age groups (>14 years vs. ≤14 years), distribution of overuse injuries by specialization level, and the relationship between rehabilitation completion and rates of subsequent injury. To assess the relationship between weekly training volume and the likelihood of reporting an overuse injury, we used logistic regression analysis, reporting odds ratios (OR) with 95% confidence intervals. All statistical tests were two-tailed, with significance defined as *p* < 0.05.

## 3. Results

### 3.1. Demographic and Anthropometric Characteristics

Among 375 eligible youth tennis players initially contacted, 256 were included in the final analysis (response rate 68.3%). Non-participants (*n* = 119) were predominantly in the youngest age categories (65.5% under 10 years) and did not differ significantly from participants in sex distribution (*p* = 0.42). The sample consisted of 153 males (59.8%) and 103 females (40.2%), with a sex ratio of 1.48. The age of the tennis players ranged from 5 to 18 years, with a median of 9 years [IQR: 8–13]. Participants were distributed across age categories as follows: Lutin (22.3%), Poussin (32%), Benjamin (15.2%), Minim (12.1%), Cadet (12.1%), and Junior (6.3%).

**Figure 2:**
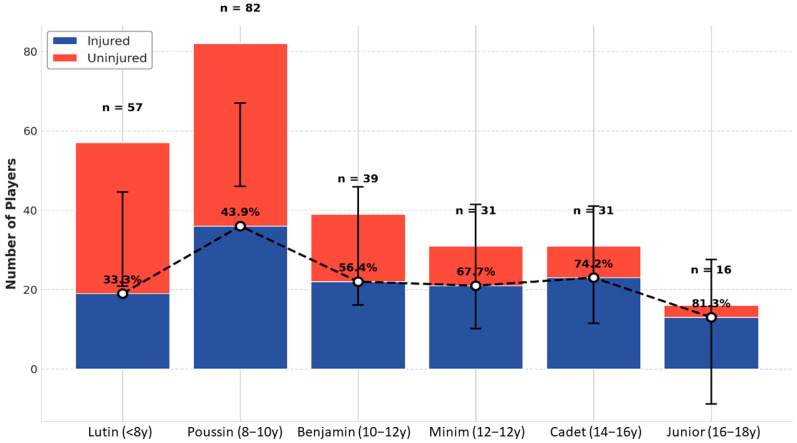
Distribution of participants by age category and injury status. Note: The dashed line indicates a progressive increase in injury frequency with age. Error bars represent 95% confidence intervals.

The median BMI (IQR) was 17.8 [15.8–20.5] kg/m^2^, with 67.6% of players having a normal BMI, 14.8% being overweight, 11.7% obese, and 5.9% underweight. Most players (84.8%) reported no significant medical history, with the most common medical conditions being allergies (8.2%) and asthma (2.3%). The percentage of players who had reached puberty was 24.6% (Table 1 and Figure 2).

### 3.2. Sporting Characteristics

The median age (IQR) for starting tennis was 5 (4.0–5.9) years, with a practice duration of 5 (3–8) years. All participants were competitive players who met the inclusion criteria, with 43% (*n* = 110) holding an official national ranking from the Tunisian Tennis Federation, representing the top 32% of ranked junior players nationally. Among the recruited players, 12.1% (*n* = 31) were involved in international competitions. Most players were right-handed (94.1%) and used a double-handed backhand (95.7%).

The weekly training regimen included a median (IQR) of 7.5 (6–8.5) h of tennis-specific practice, 2 (2–3) h of strength and conditioning, and 2 [1–2.5] h of other physical activities, yielding a tennis-to-conditioning ratio of 3.75:1 and a median (IQR) total sport exposure of 11.5 (9–14) h per week. The median (IQR) total match exposure was 72 (30–160) matches. In terms of sport specialization, 32.4% were classified as highly specialized, 65.2% as moderately specialized, and only 2.3% as lowly specialized. The median age (IQR) at which specialization began was 6 (6–7) years.

### 3.3. Injury Frequency

The frequency of injuries within our study population was 53.5%, with 137 players sustaining at least one injury during their playing period. A total of 366 injuries were reported. The median (IQR) number of injuries per player was 1 (0–2), with extremes ranging from 0 to 10. Among all players (*n* = 256), 22.3% reported one injury, 7.8% reported two injuries, and 23.4% reported three or more injuries.

The frequency of injuries increased across age categories, from 33.3% in the Lutin group to 81.3% in the Junior group. The frequency of injuries increased across age categories, from 33.3% in the Lutin group to 81.3% in the Junior group

### 3.4. Characteristics of Injuries

#### 3.4.1. Anatomical Distribution and Laterality

**Figure 3:**
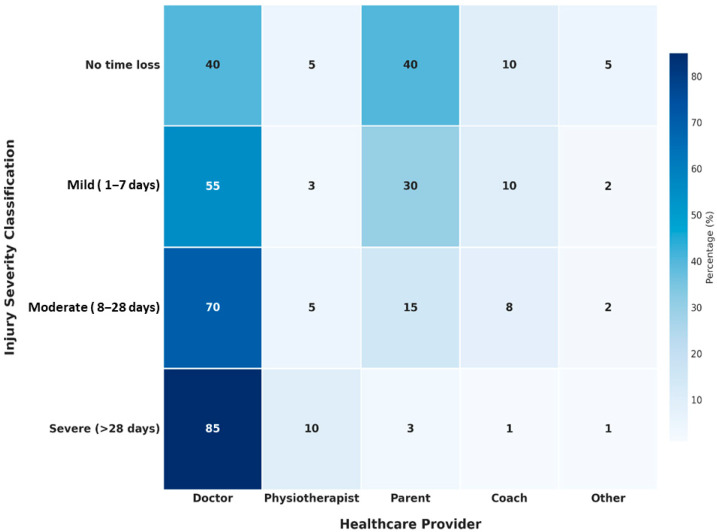
Relationship between injury severity and type of medical care received. Statistical Note: Values represent the percentage of injuries within each severity category that received care from each type of provider. Medical consultation rates increased significantly with injury severity (χ2 = 37.6, *p* < 0.001). Increasing medical professional involvement in injury severity and decreasing parental management with increasing severity. 78.8% of knee injuries received medical care, while only 35.2% received proper follow-up care. Additionally, physiotherapy utilization increased with the severity of the injury. Severe injuries (24% of cases) lack adequate rehabilitation, 64.8% of injuries received non-professional assessment, and physiotherapy services are underutilized (3.6% overall).

Injuries to the lower limb were most frequent, accounting for 58.5% of all injuries, followed by upper limb injuries (32.8%), trunk injuries (5.7%), and head and neck injuries (3%). Within the lower limb, knee injuries were the most prevalent (23.2% of all injuries), followed by ankle injuries (17.5%), thigh injuries (8.2%), and foot injuries (6.6%). For the upper limb, wrist injuries were most common (10.1%), followed by shoulder and elbow injuries (8.5% each). Right-sided injuries (66.4%) were more common than left-sided injuries (24.9%), with the remaining injuries affecting bilateral or central structures (Table 2 and Figure 3).

#### 3.4.2. Tissue Type and Mode of Onset

Muscle and tendon injuries constituted 36.9% of all cases (95% CI: 31.9–41.9), followed by superficial tissue/skin injuries (28.1%; 95% CI: 23.5–32.7) and ligament/joint capsule injuries (27.6%; 95% CI: 23.0–32.2). Muscle-tendon injuries were significantly more prevalent in older players (>14 years) compared to younger players (≤14 years) (46.3% vs. 32.9%, *p* = 0.011). Bone injuries accounted for 4.4% (95% CI: 2.4–6.4) of cases, while injuries to cartilage, synovium, and bursa represented 2.7% (95% CI: 1.1–4.3).

Regarding the mode of onset, 51.9% were acute injuries and 48.1% were repetitive injuries. Among acute injuries, 44.4% had a sudden onset (30.9% from direct contact, 6.9% from indirect contact, and 6.6% from non-contact), and 7.6% had a gradual onset. Among repetitive injuries, 28.7% had a gradual onset (all involving non-contact mechanisms), and 8.4% had a sudden onset.

#### 3.4.3. Circumstances and Severity of Injuries

Most injuries (95.9%) were directly related to tennis participation, with 74.9% occurring during practice sessions and 25.1% during competitions. The majority of injuries (93.4%) occurred in hot weather conditions. The most common mechanisms reported were changes in training intensity or method (33.9%), accidental falls (33%), overuse with unclear genesis (23%), and poor stroke technique (21.6%).

In terms of severity, 29.5% were classified as minor, 27.3% as mild, 19.1% as moderate, and 24% as severe. Four players (1.1%) reported career-ending injuries. A total of 16 bone fractures (4.4%) were recorded.

### 3.5. Overuse Injuries and Symptom Severity

#### 3.5.1. Subsequent Injury Burden and Classification

Subsequent injuries represented a substantial burden, accounting for 54.6% of all reported injuries (200/366; 95% CI: 49.5–59.7). These subsequent injuries manifested as three distinct patterns: new injuries at different anatomical locations (47.5%), recurrences of previous injuries (42%), and exacerbations of incompletely healed injuries (10.5%). The anatomical distribution of subsequent injuries predominantly affected the lower limb (63%), followed by the upper limb (31.5%).

#### 3.5.2. Rehabilitation Status and Subsequent Injury Risk

A critical relationship emerged between rehabilitation completion and subsequent injury occurrence. Players who reported incomplete rehabilitation after their index injury demonstrated a significantly higher rate of subsequent injuries compared to those who completed rehabilitation (72.3% vs. 41.5%, *p* < 0.001), highlighting the importance of comprehensive injury management protocols.

#### 3.5.3. Overuse Injury Patterns and Risk Factors

The OSTRC-O2 questionnaire identified overuse injuries in 22% of the study population, with severity distribution showing 14.5% mild problems, 6.3% moderate problems, and 1.2% severe problems. Overuse injury frequency demonstrated significant associations with training characteristics, increasing substantially with specialization level (13.9% in lowly specialized, 19.8% in moderately specialized, and 30.1% in highly specialized players; *p* = 0.022) and weekly training volume (OR = 1.18 per additional hour; 95% CI: 1.09–1.27).

### 3.6. Injury Management

Physicians assessed most injuries (57.4%), while others were evaluated by parents (24%), coaches (10.7%), or physiotherapists (3.6%). Imaging was used in 42.9% of cases, with radiography being the most common examination (89.8% of imaging studies).

Management of injuries primarily involved rest (68.9%), local treatment (64.8%), cryotherapy (42.1%), oral medication (24.9%), and rehabilitation (27.9%). Orthopedic management, such as casting or splinting, was used in 5.5% of cases. Good therapeutic adherence was noted in 78.7% of injuries.

An evaluation of physical condition and performance was conducted in 35.5% of cases before reintegration. Players underwent progressive reintegration into training and matches after injury in 40.7% of cases. Only 35.2% of injuries received medical follow-up care.

## 4. Discussion

This study represents the first epidemiological investigation of tennis injuries among youth competitive players in Tunisia, a region previously underrepresented in sports injury research despite its growing tennis participation rates and unique environmental conditions. Our findings reveal a substantial injury burden, with 53.5% of players reporting at least one injury during their tennis career, consistent with international data showing injury frequencies of 46–54% in similar populations [23,24]. The injury incidence rate of 7.8 injuries per 1000 player-hours aligns with rates reported among Dutch (7.1/1000 h) and British (6.9/1000 h) junior players [2,12].

The anatomical distribution demonstrates clear lower limb predominance (58.5%), with knee injuries accounting for 23.2% of all injuries. This pattern reflects tennis-specific biomechanical demands, where players experience more than eight directional changes per point occurring every 1.5 s, applying loads of 1.5 to 2.7 times body weight on knee structures [23]. While these data [23] partly involve professional athletes, the fundamental movement patterns are similar in youth players, making them relevant for understanding injury mechanisms. However, given growth-related vulnerabilities, these loads may pose greater risks for developing musculoskeletal structures in younger athletes, emphasizing the need for age-appropriate prevention strategies. Ankle injuries (17.5%) similarly reflect the sport’s dynamic nature, involving rapid directional changes, predisposing to both acute sprains and chronic conditions [25].

Upper limb injuries affecting the wrist (10.1%), shoulder (8.5%), and elbow (8.5%) regions demonstrate patterns consistent with repetitive, high-intensity stroke mechanics. However, the relative distribution differs from Western cohorts, where shoulder injuries typically predominate [6], potentially reflecting regional differences in playing style, equipment selection, or coaching methodologies. Although this study [6] predominantly includes adult or professional players, these data provide relevant contextual information as the fundamental biomechanics of tennis strokes are broadly comparable across age groups. Nonetheless, developmental differences in musculoskeletal maturity, neuromuscular control, and cumulative training exposure may alter injury susceptibility in youth athletes, which should be considered when interpreting these comparisons.

Wrist injuries result from repetitive motions in backhand and serve strokes [26], while shoulder pathology often involves rotator cuff dysfunction associated with joint instability in youth athletes [27]. Elbow injuries, particularly lateral epicondylitis, commonly result from repetitive wrist extension during backhand groundstrokes [27].

Our finding that muscle-tendon injuries represented the predominant tissue type affected (36.9%) is consistent with established tennis injury patterns [24]. The distribution between acute (51.9%) and repetitive injuries (48.1%) aligns with elite junior cohort studies [27], though it contrasts with some data suggesting chronic injury predominance [12]. This discrepancy may reflect methodological differences, underreporting of gradual onset injuries not causing immediate time loss, or regional reporting variations. The OSTRC-O2 questionnaire implementation enabled identification of overuse injuries in 22% of participants, which traditional time-loss definitions would have missed [20].

The finding that 93.4% of injuries occurred in hot weather conditions is particularly relevant for Tunisia’s Mediterranean climate, featuring prolonged high-temperature periods from May to September. Youth athletes under these conditions may experience dehydration and heat-related physiological stress, which recent evidence suggests impairs neuromuscular control and proprioception, thereby increasing injury risk [13,28]. Most injuries occurred during practice sessions (74.9%) rather than competitions (25.1%), consistent with previous studies reporting 56.2–76% practice-related injury occurrence [29,30], reflecting greater training exposure and indicating that prevention strategies should prioritize training environments.

The healthcare utilization patterns reveal concerning gaps in sports medicine access, with only 57.4% of injuries assessed by physicians and merely 3.6% evaluated by physiotherapists. This contrasts markedly with Western European data showing 68–75% physiotherapy consultation rates for significant junior tennis injuries [2]. Similarly, low rehabilitation utilization (27.9%) and medical follow-up rates (35.2%) indicate substantial healthcare system gaps compared to Brazilian junior players, where 58.1% sought medical assistance [24].

The severity profile demonstrates concerning patterns, with 24% of injuries classified as severe and only 29.5% as minor, comparable to Llanes et al. [29], who reported 14% of players quitting sport due to injury. Severe injuries may particularly impact youth development in resource-limited settings where training absence significantly affects competitive progression.

Most critically, subsequent injuries accounted for 54.6% of all injuries, substantially exceeding rates reported in similar studies (9–35%) [24,29,30]. This elevated recurrence rate likely reflects inadequate rehabilitation, premature return-to-play decisions, or insufficient injury surveillance systems. O’Connor et al. [24] identified previous injury as a significant reinjury risk factor, emphasizing the importance of complete rehabilitation before returning to sports. The significantly higher subsequent injury proportion compared to higher-resourced nations suggests a potential breakdown in the injury management continuum, possibly reflecting sociocultural attitudes toward injury, economic constraints limiting specialized care access, and an absence of formalized return-to-play protocols [20].

The underutilization of rehabilitation services (27.9%) and structured return-to-play protocols (40.7%) represents a significant intervention opportunity. Evidence demonstrates that structured neuromuscular training programs reduce youth sports injury rates by 30–40% [28], while standardized return-to-play protocols decrease subsequent injury rates by up to 50% [28]. These findings highlight the critical need for comprehensive sports medicine infrastructure development and evidence-based injury prevention program implementation in developing tennis markets.

### 4.1. Limitations

Several methodological limitations warrant consideration when interpreting the findings of this investigation. First, recall bias is inherent in retrospective injury reporting, particularly for injuries that occurred in the more distant past. While we attempted to minimize this bias by involving parents in the data collection process, minor injuries may still have been underreported. Second, injury data were based on self-reports by players and their parents, without systematic clinical verification, which may have affected the accuracy of injury classification. We attempted to address this gap by examining the clinical presentation, reviewing the imaging results, and managing the injury. Third, our study population was limited to competitive players from three regions in Tunisia, which may limit the generalizability of our findings to recreational players or those from other areas with different environmental conditions or training practices. Fourth, the assessment of pubertal status was based on self or parent-reported responses, without clinical or hormonal confirmation. This method was chosen due to logistical and ethical constraints, especially in the context of an extensive cross-sectional field study conducted in sports club settings. While practical in a field-based setting, this subjective method may have led to misclassification, limiting the accuracy of maturity-related analyses. Finally, the training exposure calculation was based on weekly estimates multiplied by practice duration, which may not accurately reflect variations in training volume over time. Additionally, the use of BMI classifications based on WHO growth charts, although standardized, may not optimally reflect body composition in youth athletes with sport-specific anthropometric adaptations [17]. Future research should emphasize prospective study designs that incorporate systematic clinical verification to enhance the accuracy and reliability of injury data. Such approaches will allow for more precise injury classification and a better understanding of injury mechanisms in this population.

### 4.2. Practical Applications

Based on our findings, we recommend the following: (i) implementation of targeted neuromuscular training programs focusing on lower limb injury prevention; (ii) development of accessible rehabilitation pathways for injured youth players; (iii) education of coaches, parents, and players regarding appropriate injury management and gradual return-to-play progressions; and (iv) integration of regular preparticipation screening to identify modifiable injury risk factors. These measures have the potential to significantly reduce the tennis injury burden in this previously understudied population.

## 5. Conclusions

This study provides the first epidemiological overview of tennis-related injuries among youth competitive players in Tunisia. Based on these findings, several practical implications emerge. Injury reduction should be addressed through the establishment of structured surveillance systems, the integration of evidence-based neuromuscular training in regular practice, and improved access to sports medicine and rehabilitation services. Effective prevention requires coordinated efforts among coaches, parents, and healthcare professionals to ensure appropriate load management, early injury detection, and safe return-to-play decisions. At a policy level, sports federations and decision-makers in North Africa should allocate resources to injury prevention education and rehabilitation programs adapted to local socioeconomic and environmental conditions. Future research should focus on prospective studies with standardized surveillance protocols to identify context-specific risk factors and evaluate culturally tailored prevention strategies. Implementing these measures could enhance both the long-term health and athletic performance of youth tennis players in the region.

## Figures and Tables

**Table 1:** Sociodemographic and medical data of Tunisian youth tennis players (*n* = 256).

Variables	Characteristics	*n*	%
Age category	Lutin	57	22.3
	Poussin	82	32
	Benjamin	39	15.2
	Minim	31	12.1
	Cadet	31	12.1
	Junior	16	6.3
Sex	Boy	153	59.8
	Girls	103	40.2
Medical history	None	217	84.8
	Allergy	21	8.2
	Asthma	6	2.3
	Other conditions	12	4.7
Body mass index class	Overweight	38	14.8
	Obese	30	11.7
	Normal	173	67.6
	Underweight	15	5.9
Puberty	Yes	63	24.6
	No	193	75.4

**Table 2:** Distribution of injuries by anatomical location among Youth Tunisian Tennis (*n* = 366).

Body Region	Body Area	Body Side				Total	
		Right		Left		*n*	%
		*n*	%	*n*	%		
Head and neck	Head					9	2.5
	Neck					2	0.6
	Total					11	3
Trunk	Chest					2	0.6
	Spine					15	4
	Abdomen					4	1
	Total					21	5.7
Upper Limb	Shoulder	29	7.9	2	0.6	31	8.5
	Upper Arm	1	0.3	0	0	1	0.3
	Elbow	24	6.6	7	1.9	31	8.5
	Forearm	1	0.3	0	0	1	0.3
	Wrist	29	7.9	8	2.2	37	10.1
	Hand	13	3.6	6	1.6	19	5.2
	Total	97	26.5	23	6.3	120	32.8
Lower Limb	Hip/groin	4	1	0	0	4	1.1
	Thigh	22	6	8	2.2	30	8.2
	Knee	52	14.2	33	9	85	23.2
	Lower leg	7	1.9	0	0	7	1.2
	Ankle	46	12.6	18	4.9	64	17.5
	Foot	15	4.1	9	2.5	24	6.6
	Total	146	39.9	68	18.6	214	58.5
Total		243	66.4	91	24.9	366	100

## Data Availability

The raw data supporting the conclusions of this article will be made available by the authors on request.
